# What Healthcare Workers Should Know about Environmental Bacterial Contamination in the Intensive Care Unit

**DOI:** 10.1155/2017/6905450

**Published:** 2017-10-29

**Authors:** Vincenzo Russotto, Andrea Cortegiani, Teresa Fasciana, Pasquale Iozzo, Santi Maurizio Raineri, Cesare Gregoretti, Anna Giammanco, Antonino Giarratano

**Affiliations:** ^1^Department of Biopathology and Medical Biotechnologies (DIBIMED), Section of Anesthesia, Analgesia, Intensive Care and Emergency, University Hospital Policlinico Paolo Giaccone, University of Palermo, Palermo, Italy; ^2^Department of Sciences for Health Promotion and Mother and Child Care, University of Palermo, Palermo, Italy

## Abstract

Intensive care unit- (ICU-) acquired infections are a major health problem worldwide. Inanimate surfaces and equipment contamination may play a role in cross-transmission of pathogens and subsequent patient colonization or infection. Bacteria contaminate inanimate surfaces and equipment of the patient zone and healthcare area, generating a reservoir of potential pathogens, including multidrug resistant species. Traditional terminal cleaning methods have limitations. Indeed patients who receive a bed from prior patient carrying bacteria are exposed to an increased risk (odds ratio 2.13, 95% confidence intervals 1.62–2.81) of being colonized and potentially infected by the same bacterial species of the previous patient. Biofilm formation, even on dry surfaces, may play a role in reducing the efficacy of terminal cleaning procedures since it enables bacteria to survive in the environment for a long period and provides increased resistance to commonly used disinfectants. No-touch methods (e.g., UV-light, hydrogen peroxide vapour) are under investigation and further studies with patient-centred outcomes are needed, before considering them the standard of terminal cleaning in ICUs. Healthcare workers should be aware of the role of environmental contamination in the ICU and consider it in the broader perspective of infection control measures and stewardship initiatives.

## 1. Introduction

Intensive care unit- (ICU-) acquired infections are a major health problem worldwide. Emergence of multidrug resistant organisms (MDROs) poses a daily challenge to ICU physicians, dealing with critically ill patients with multiple risk factors for infections (i.e., impairment of body barriers due to invasive devices and surgery, immunosuppression, prolonged antibiotic exposure) [1–4]. A relevant body of evidence highlights the high prevalence of contamination of high-touch surfaces and equipment surrounding patients' bed [5]. Indeed, the patient's nearby environment is crowded by equipment for monitoring and organ support (e.g., monitors, ventilator, extracorporeal life support machines), requiring sophisticated and specific cleaning procedures. Contamination of inanimate surfaces may occur as the consequence of direct patient shedding of bacteria (higher from infected than colonized patients) or via healthcare workers' (HCWs') hands. HCWs contaminate their hands from inanimate surfaces as frequently as direct patient contact [6]. In a randomized cross-over study, recontamination of high-touch surfaces in ICU occurred after only 4 hours from standard cleaning measures [7]. Environmental contamination in the ICU involves not only equipment for direct patient care (e.g., stethoscopes, ultrasound equipment, surfaces of mechanical ventilators) but also surfaces of objects used for clinical data recordings (i.e., medical charts, computer keyboard, and mouse) and mobile phones [8]. Environmental contamination has been identified as a major contributor of bacteria cross-transmission and patient colonization and infection. In 1991, Weinstein [9] estimated the relative contribution of different potential sources for ICU-acquired infections: 40–60% patient's endogenous flora, followed by cross-infection via HCWs' hands (20–40%), antibiotic-driven changes in flora (20–25%), and other sources (including environmental contamination, 20%). Understanding the mechanisms underlying cross-transmission of pathogens from inanimate surfaces and equipment may contribute to lay the foundation of effective infection control measures aiming to halt the spread of healthcare-associated infections. The aim of this review is to provide updated evidence on environmental contamination in the ICU, focusing on mechanisms by which bacteria are able to survive on inanimate surfaces, describing the concept of patient zone and healthcare area and the role of contamination for ICU-acquired colonization and infection.

### 1.1. The Concepts of Patient Zone and Healthcare Area

The concepts of* patient zone* and* healthcare area* have been introduced as a user-centred, operative behaviour aiming to enhance hand hygiene compliance [10]. The patient zone encompasses the patient and surfaces and equipment surrounding him/her (i.e., bed rails, ventilator, monitors). The healthcare area is composed of all surfaces outside a given patient zone (i.e., the healthcare facility environment and other patient zones) [8].

The healthcare area may be contaminated by bacteria from different patient zones. Inanimate surfaces in the patient zone are contaminated by bacteria colonizing/infecting patients in two ways: direct shedding from patients and via HCWs' hands. High-touch objects in the immediate vicinity of patients are heavily contaminated. A higher degree and rate of contamination occur from infected patients than from patients who are only colonized. Moreover, a correlation exists between number of culture-positive body sites and environmental contamination [11, 12]. A high degree of patient zone contamination has been reported also in case of patients with diarrhea [13, 14].

**Figure 1:**
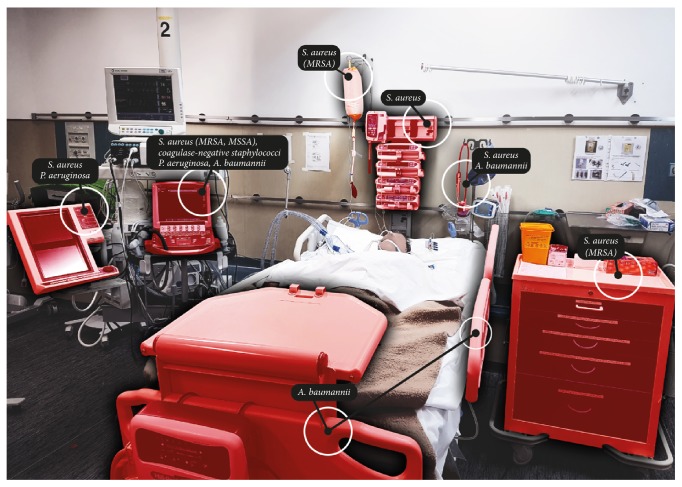
Patient zone with more frequently isolated bacteria contaminating inanimate surfaces and equipment.

Figure 1 shows a patient zone with most frequently reported contaminating bacteria in the literature.

### 1.2. ICU-Acquired Colonization and Infection: Update on Available Evidence

Evidence on the role of environmental contamination for cross-transmission of pathogens comes from studies reporting on outbreaks of infections driven by contaminated objects or equipment, studies investigating the association of colonized/infected patients with environmental and HCWs' hands contamination, and studies reporting on the risk of acquiring bacteria from prior bed occupants [15]. Hand-washing sinks, bottled still water, and bronchoscope suction valves have been related to outbreaks registered in ICUs, with the same strains and antibiotic susceptibility profiles registered in those isolated from colonized/infected patients [16–21]. This observation is of value when we consider the role of inanimate surfaces contamination as a reservoir of MDROs of potentially pathogen role.

In their cohort study, Morgan et al. [22] investigated how frequently HCWs contaminated gloves and gowns after contact with patients. Approximately, after one of every three interactions with a patient carrying* Acinetobacter baumannii*, HCWs contaminated their gloves and gowns.* A. baumannii* was present in almost 80% of rooms from colonized patients. Contamination with* A. baumannii* occurred more frequently than with other bacteria (*Pseudomonas aeruginosa*, vancomycin-resistant Enterococci and methicillin-resistant* Staphylococcus aureus*). Independent risk factors for HCWs contamination by MDROs were positive environmental cultures (OR 4.2, 95% CI 2.7–6.5), stay in room for more than 5 minutes (OR 2.0, 95% CI 1.2–3.4), performing physical examination (OR 1.7, 95% CI 1.1–2.8), and contact with the ventilator (OR 1.8, 95% CI 1.1–2.8) [22].

A number of studies reported on a higher risk of acquiring bacteria from prior room occupants. This independent risk factor occurred for both Gram-positive* (S. aureus, Enterococcus species, Clostridium difficile)* and Gram-negative bacteria* (Acinetobacter *spp.*, P. aeruginosa, Klebsiella pneumoniae)* [23], including MDROs (MRSA, VRE). We recently performed a meta-analysis of studies investigating this issue in the ICU setting [24]. The pooled OR of acquisition of bacteria from prior bed occupants was 2.13 (95% CI 1.62–2.81). When we considered the OR for the acquisition according to bacterial species, we registered the highest OR for* A. baumannii* (OR 4.91, 95% CI 2.79–8.64) and* C. difficile* (OR 2.57, 95% CI 1.28–5.15). It is remarkable that this increased risk occurs even when current terminal cleaning procedures are addressed. We may speculate that these findings may be explained by suboptimal terminal cleaning procedures resulting in persisting surfaces contamination. Environmental contamination may represent the reservoir for cross-transmission of bacteria via HCWs' hands. Structural ICU features may be associated with a different degree of environmental contamination and cross-transmission rate. Indeed, single-room ICUs have the theoretical advantage of a physical separation of different patient zones. This may be also associated with the ease of adoption of enhanced terminal cleaning procedures requiring environment isolation. However, cross-transmission of bacteria from prior bed occupant occurred in single-room ICUs in most studies [25].

### 1.3. Terminal Cleaning in ICU

The term* terminal cleaning* refers to all methods used for disinfection of either a room or a patient zone between occupying patients (i.e., after patient discharge). Quaternary ammonium and bleach are the most commonly used products for this purpose. The efficacy of terminal cleaning relies on different factors, including training and management of personnel (e.g., adequate contact time, compliance with protocols) and accessibility of surfaces. If we consider the higher patient's risk of acquiring a MDRO if exposed to the bed of a previously colonized/infected patient, current terminal cleaning methods are far from being considered a highly effective procedure. Inadequate cleaning as assessed by objective measures of operators' performance has been reported in different studies. Programs including a training phase, followed by an objective monitoring of operators' performance through the use of assays such as adenosine triphosphate (ATP) bioluminescence, resulted in an overall improvement of performance and reduced degree of environmental contamination [26, 27]. In a prospective study [28], authors investigated a bundle of interventions aiming to enhance room cleaning in ICUs after patient discharge. They collected baseline cleaning performance using a mark visible only with an ultraviolet lamp (black-light indicator). Study interventions consisted of an increased disinfectant volume application, an educational campaign involving the cleaning staff and the adoption of a black-light monitoring system for feedback. The indicator was removed from 72% of surfaces after the interventions, compared to 44% cleaned surfaces at baseline (*p* < 0.001). The interventions also reduced the environmental positive cultures for MRSA and VRE [28]. Similar results were reported in another prospective study conducted in 27 ICUs where only 49.5% of selected surfaces were cleaned at baseline, compared to 82% after a structured educational, procedural, administrative intervention and objective feedback provided by a fluorescent marker [29].

In 2010 the Centres for Disease Control and Prevention provided a toolkit with a bundle of interventions for improvement of hospital cleaning. Objective assessment has been incorporated among the interventions to apply to enhance cleaning performance. The proposed methods were direct cleaning practice observation, swab and agar slide cultures, fluorescent markers, and ATP bioluminescence [30]. Recently, no-touch terminal cleaning procedures have been investigated in ICUs. They consist of the use of either physical (e.g., ultraviolet, UV-light) or chemical agents (e.g., hydrogen peroxide, H_2_O_2_ vapour, HPV) delivered by specific devices without the active role of personnel. The theoretical advantages of these techniques are a higher overall performance given the capability to disinfect difficult to reach surfaces, difficult to clean equipment, and the lack of reliance on personnel performance.

UV-light generates DNA and RNA alterations leading to an irreversible damage and killing of microorganisms. The device is normally placed in the room centre and it is activated by remote control. Surfaces receiving direct irradiation are exposed to the highest killing potential but wall, floor, and other surfaces are able to reflect the UV-light and indirectly expose other surfaces. However, shadowed areas may not receive a sufficient radiation dose and UV-light does not penetrate porous surfaces. Two different UV-power levels are available for killing of vegetative and spore-forming pathogens. Different studies reported the high capacity of UV radiation to significantly reduce the contamination of high-touch surfaces by a number of pathogens (MRSA, VRE and* C. difficile*) [31, 32]. Compared to HPV, UV-light decontamination is cheaper to operate and maintain and requires less time [33]. Anderson et al. [34] recently performed a large (31226 exposed patients) multicentre randomized study investigating the adoption of enhanced terminal cleaning procedures of seed rooms by previously colonized/infected patients. They compared 4 different strategies: UV-light combined with quaternary ammonium, UV-light combined with bleach, bleach, and reference (quaternary ammonium). Notably, according to their standard cleaning protocol, bleach was used as reference for rooms seed by* C. difficile*. Adding UV-light led to a reduction of the incidence of colonization and infection caused by MRSA and VRE, while authors did not observe a statistically significant difference when UV-light was compared to bleach. Of note, only one outcome occurred for* Acinetobacter* and therefore the role of enhanced terminal cleaning was not investigated for this pathogen. The lack of benefit for* C. difficile* cross-transmission was explained by the adoption of an enhanced procedure also in the reference group (i.e., bleach), the high compliance of personnel to the cleaning protocol (which may be significantly lower in real life), and the use of a single-stage cycle of UV-light for a pathogen with a time- and dose-dependent response to UV-light. Despite these limitations, this is the first trial to date enrolling such a high number of patients and adopting the clinically relevant outcomes of infections and colonization [35].

H_2_O_2_ is a noncorrosive agent showing bactericidal, fungicidal, sporicidal, and virucidal properties in vitro. H_2_O_2_ damages lipid membranes, DNA, and RNA through its oxidative action. HPV showed effective decontamination against MRSA, VRE,* Acinetobacter *spp.,* K. pneumonia*, and* C. difficile* spores [36]. H_2_O_2_ may be released in three different forms: dry vapour, wet vapour, and mist. These three technologies produce H_2_O_2_ particles small enough to diffuse and reach hidden and difficult to reach surfaces. Recently, Blazejewski et al. [37] investigated the efficiency of HPV in improving disinfection in ICUs. They applied HPV after routine terminal cleaning and collected environmental sampling before and after HPV use. After patient discharge, 8% of sampled rooms were contaminated with at least 1 MDRO. Routine terminal cleaning reduced the environmental bacterial load but authors did not detect a statistically significant difference in the degree of MDROs contamination [37]. HPV, instead, significantly reduced the residual environmental contamination by MDROs. Given the high costs for implementation of no-touch terminal cleaning methods in ICUs (i.e., machines and maintenance costs, additional staff members), further studies are needed to evaluate their impact for patient-centred outcomes.

## 2. How Bacteria Survive on Inanimate Surfaces and after Terminal Cleaning Procedures?

The principal factors associated with the ability of a nosocomial pathogen to survive on inanimate surfaces and equipment are the specific microorganism characteristics (such as genus, species, specific strain, ability to form biofilm, and microorganism concentration) and the environmental factors (such as UV radiation, temperature, humidity, presence of organic materials, and surface type) [38–40]. Evidence on the capacity to survive in environmental reservoirs has been reported for bacteria (*C. difficile*, VRE, MRSA,* P. aeruginosa, Escherichia coli, Klebsiella *spp., and* Acinetobacter *spp.), viruses (influenza, parainfluenza, enteric, hepatitis B viruses), and fungi (*Candida albicans*,* Candida glabrata, Candida parapsilosis*,* Aspergillus *spp., and Zygomycetes) [41–47]. Microorganisms are able to survive on surfaces because of their production of adhesion molecules and biofilms. These abilities are favoured when microorganisms grow on materials with high absorptive capacity [48]. Coagulase-negative staphylococci are able to survive up to 8–21 days on cotton used to produce clothing and towels, while* P. aeruginosa* survives for only 2–24 hours on the same surface. Even different species from the same genus showed different survival capacity [42]. As an example,* C. parapsilosis* showed higher resistance when compared to* C. albicans* or* C. krusei *[49]. Intrinsic microbiologic features also influence the resistance against disinfectants. For example, mycobacteria have a waxy cell wall able to prevent disinfectants entry, whereas Gram-negative bacteria have an outer membrane acting as a barrier preventing the uptake of disinfectants [50]. Concentration of Gram-positive and Gram-negative bacteria, fungi, and viruses may influence their persistence on surfaces; the greater the microbial load, the longer the survival capacity. A biofilm is a structured community of microorganisms encased and attached to surfaces by exopolymeric substances (EPS). Up to 90% of biofilm are made of EPS, which provides protection against environmental desiccation. Biofilm plays an important role in catheter-related infections and of other indwelling medical devices [51]. Bacteria are able to form biofilm also on dry inanimate surfaces. It has been speculated that biofilm formation may be enhanced by a thin film of water resulting from condensation on surfaces or that the relative humidity of ICUs is sufficiently high to allow biofilm formation [52]. Biofilms contain a high bacterial load able to survive on dry hospital surfaces for a long time, showing also an increased resistance towards inactivation by disinfectants. Indeed, bacteria in the biofilm are up to 1000-fold more resistant to disinfectants than their corresponding planktonic form [40, 52–55].* P. aeruginosa *biofilm on flexible endoscopes surfaces is able to survive 5-minute treatment with peracetic acid at 2000 parts per million concentration, which is the working concentration used by some washer-disinfectors [54].

Vickery et al. [52] investigated the persistence of reservoirs of MDROs within biofilm after terminal cleaning in an ICU. Equipment and furnishings were aseptically removed from the ICU, scanned by electron microscopy and cultured. Biofilm was demonstrated on 4 of the five different samples from the patient zone and healthcare area. Cultures from samples led to MRSA growth. This finding highlights one possible explanation of the suboptimal terminal cleaning efficacy and the persistence of a reservoir of MDROs possibly involved in direct or indirect (via HCWs' hands) cross-transmission [52]. This should be considered in the broader perspective of emergence of MDROs, stewardship initiatives, and infection control measures [45, 56–58].

The increased resistance of biofilms to disinfectants is supposed to be due to the following factors:

- gene regulation of microorganisms with increased lateral gene transfer and mutation rates [59];
- phenotypic adaptation of cells to sublethal disinfectant concentration [60];
- production of EPS surrounding the bacteria. The EPS reduces the penetration of biocides into the biofilm, inactivates some disinfectants by binding to them, and inactivates some disinfectants by liberation of enzymes [61];
- biofilm which can be composed by different microorganism species constituting a polymicrobial biofilm with a higher resistance to disinfectants compared to monospecies biofilms [61]. The mechanism of this increased resistance may result from an increased disinfectant inactivation due to a more complex EPS or from shielding of sensitive organisms by externally situated disinfectant tolerant organisms [62].

Among environmental variables, ultraviolet (UV) light, temperature, humidity, and presence of organic material have been reported to have a major role in influencing microbial viability. Visible light and UV radiation are generally deleterious to microorganisms. Temperatures higher than 50°C are able to kill most* Candida *spp. while low temperatures (4°C to 6°C) increase survival times for many bacteria. Humidity can have variable effects on the persistence of microorganisms on surfaces. Yeast showed a better survive at higher humidity [42]. Organic matter (e.g., blood, serum, sputum, pus, fecal material) may play both a direct and an indirect role to enhance environmental resistance of microorganisms. The direct role is the barrier effect protecting microorganisms from environmental physical and chemical agents. The indirect role is the interference of organic matter with the antimicrobial activity of disinfectants through chemical reactions resulting in a complex exhibiting less germicidal or nongermicidal properties and leaving a reduced quantity of active disinfectant agents. This frequently occurs with chlorine and iodine disinfectants [63]. In parallel with development of new strategies to enhance disinfectant agents' efficacy, different research groups are now focusing on development of novel materials which may potentially be used to prevent or reduce contamination and biofilm formation by bacteria, including MDROs. Metal-embedded surfaces (copper, gallium, and titanium) were effective at preventing planktonic and biofilm growth of* P. aeruginosa, S. aureus*, and* E. coli *tested strains [64]. In a recently published observational study, silver-embedded screens used to separate ICU beds were more effective than traditional cloth screens at reducing surfaces contamination and cross-transmission of pathogens [65].

## 3. Conclusions

Inanimate surfaces and equipment contamination play a major role in cross-transmission of pathogens in ICUs. Bacteria, including MDROs, may survive for a long time to environmental physical and chemical agents and have been isolated from different surfaces and equipment of the patient zone and of the healthcare area [8]. Traditional terminal cleaning methods showed major flaws and no-touch methods are under investigation [24, 34]. Clinicians should be aware of the issue of environmental contamination and consider it in the broader perspective of infection control measures and stewardship initiatives [58].
